# Self-Regulating Polymer–Carbon Nanotube Composite Heater on Meta-Aramid Textile

**DOI:** 10.3390/polym18192315

**Published:** 2026-09-22

**Authors:** Ha Eun Kang, Ji-Hyeok Choi, Seung Hyun Jee, Dong-Joo Kim, Sun Hee Kim

**Affiliations:** 1 Department of Materials Science and Engineering, Gachon University, 1342 Seongnam-daero, Sujeong-gu, Seongnam 13120, Republic of Korea; 2 Mcell Co., Ltd., 112, 10, Pangyodaejang-ro 8-gil, Bundang-gu, Seongnam 13544, Republic of Korea; 3 Materials Research and Education Center, Auburn University, 275 Wilmore Labs, Auburn, AL 36849, USA; 4 Department of Fashion Industry, Incheon University, Incheon 22012, Republic of Korea

**Keywords:** meta-aramid fiber, PTC ink, carbon nanotube (CNT), composite heater, self-regulation, electrothermal behavior, flame retardancy, polymer composite heater

## Abstract

Electric vehicles (EVs) lack a substantial source of recoverable powertrain waste heat, making cabin heating a direct parasitic load on the battery and thereby increasing the need for efficient and spatially distributed thermal management strategies. In this study, a flexible self-regulating surface heater was developed by integrating a polymeric positive temperature coefficient (PTC) multi-walled carbon nanotube (MWCNT) composite with a flame-resistant meta-aramid textile substrate. The principal contribution of this work is the systematic control of the PTC:CNT ink ratio to correlate CNT-mediated conductive-network formation with the average temperature coefficient of resistance (TCR) and electrothermal self-regulation, together with its implementation in a thermally robust textile architecture for elevated-temperature operation. Among the investigated ink ratios of 95:5–80:20, the 90:10 formulation provided the most favorable balance between electrical conduction and PTC response, reaching a stable temperature of approximately 130 °C at 24 V without thermal runaway. The optimized heater retained stable electrical functionality after repeated bending and washing, while the meta-aramid substrate provided improved resistance to ignition under severe thermal exposure. A cabin-mimicking chamber experiment further demonstrated the system-level heating capability of the textile heater under controlled power-input conditions. These results demonstrate that composition-controlled PTC-CNT network engineering on thermally robust textiles provides a viable approach to flexible and self-regulating heating systems for EV thermal management.

## 1. Introduction

The rapid deployment of electric vehicles (EVs) has become a key driver for achieving carbon neutrality and sustainable mobility [1,2]. Unlike internal combustion engine vehicles, however, EVs generate negligible waste heat from the powertrain, and thus a substantial portion of the onboard electrical energy must be directly consumed for cabin heating [3]. This additional heating demand directly competes with the energy required for vehicle propulsion and can substantially reduce the driving range, particularly under cold ambient conditions [4]. Therefore, reducing cabin-heating energy demand while delivering heat efficiently to occupants remains a key challenge in EV thermal management [5,6,7]. This challenge becomes particularly significant at low ambient temperatures, where heating, ventilation, and air-conditioning (HVAC) operation can substantially increase auxiliary energy consumption and reduce the available driving range.

In current commercial EVs, cabin heating is predominantly provided by ceramic PTC heater modules based on BaTiO_3_-type PTC ceramics [8]. These ceramic PTC heaters offer rapid thermal response and inherent self-regulating behavior in the high-temperature regime [9]; however, they suffer from high power consumption per unit area and considerable heat loss along the air duct, which limit their overall energy efficiency [10]. In addition, the brittle and rigid nature of ceramic elements makes it difficult to implement them as large-area surface heaters conformable to complex geometries such as seats, door trims and dashboard panels. Conventional ceramic PTC elements are also generally incorporated into forced-air heating modules, making direct integration into curved or distributed surface-heating structures difficult. These limitations motivate the development of flexible, distributed heating systems capable of providing localized heat closer to occupants while retaining intrinsic self-regulating behavior. Consequently, there is a growing demand for flexible surface-heating textiles that can complement conventional ceramic PTC heater modules through mechanically compliant and spatially distributed heat delivery [11,12,13,14].

Meta-aramid (m-aramid) fibers have attracted increasing attention as next-generation substrates for such heating elements because they combine high thermal stability, excellent flame retardancy, electrical insulation and mechanical flexibility [15,16]. M-aramid textiles remain thermally stable above 400 °C and can be readily processed into woven or nonwoven forms, providing a high degree of design freedom for large-area and curved heating structures. Their electrically insulating, yet ink-coatable surface enables stable deposition of polymer-based functional inks while maintaining adhesion and electrical integrity under repeated bending and washing [17,18]. These features make m-aramid fabrics a promising platform for flexible electronics and textile-based heating systems.

CNT-containing aramid structures have also been investigated for flexible electrothermal applications. MWCNT–aramid composite films and CNT-coated aramid structures have demonstrated that conductive carbon networks can be incorporated into thermally robust aramid frameworks while retaining mechanical flexibility [16,19]. However, these systems have primarily operated as conventional resistive joule heaters, with their equilibrium temperature governed mainly by applied voltage, electrical resistance, and heat dissipation.

Polymer-based PTC materials offer additional advantages over ceramic PTCs in terms of processing temperature and printability [11,18,20,21]. PTC105, used in this study, is a polymeric PTC ink consisting of a polymer matrix filled with PTC-active particles, exhibiting a characteristic increase in resistance with temperature [22]. However, polymer PTC-based materials alone often struggle to simultaneously satisfy the requirements of a target self-regulation temperature, sufficient electrical conduction, and controlled temperature-dependent resistance variation [21]. To overcome this limitation, the incorporation of conductive nanofillers is required to tailor the temperature-dependent resistance and optimize the electrothermal response [18,23].

Carbon-based nanomaterials such as carbon nanotubes (CNTs) possess exceptionally high electrical and thermal conductivity and can play a crucial role in forming conductive networks and tuning the PTC response in composite inks [24,25,26,27]. CNT-filled composite PTC inks can reduce the initial resistance by providing additional current pathways and moderate the resistance increase during heating, thereby enabling the design of self-heating behavior that approaches a desired target temperature [18]. As the temperature increases, thermal expansion of the polymer matrix can alter the distance and contact state between neighboring CNTs, thereby modifying tunneling conduction and conductive-network connectivity [28]. Consequently, the temperature-dependent resistance response is strongly influenced by both CNT concentration and the resulting network structure. By carefully controlling the CNT loading and dispersion state [29], it is possible to engineer an ideal PTC response in which stable self-regulation is achieved without thermal runaway, making such systems highly attractive for surface-heating applications in EV seats and interior components [18].

More directly relevant studies have demonstrated that CNT concentration and conductive-network configuration strongly influence the temperature-dependent electrical response of polymer composites.

Kil et al. showed that changes in CNT content and network structure can determine whether CNT–polypropylene composites exhibit positive or negative temperature coefficient behavior during self-heating [30]. This finding indicates that increasing CNT loading does not simply enhance electrical conduction, but can also modify the self-regulating characteristics of the composite.

In aramid-based systems, Jeong et al. reported that increasing MWCNT content in MWCNT–m-aramid composite films promoted the formation of a three-dimensional conductive network, reduced electrical resistivity, and increased the attainable joule-heating temperature [31]. However, their system primarily functioned as a conventional resistive heater rather than through a polymeric PTC self-regulation mechanism. PTC-MWCNT composite pastes have also been incorporated into textile heaters for self-regulating wearable-heating applications [17,18,31], however, these studies generally targeted substantially lower operating temperatures than those considered for EV-oriented surface heating.

Thus, although CNT-based flexible heaters and PTC-CNT self-regulating composites have been investigated separately, the combined optimization of CNT-mediated electrical conduction, PTC self-regulation, flame-resistant textile integration, and higher-temperature operation for EV cabin heating remains insufficiently addressed.

In this paper, we report a self-regulating polymer–CNT composite heater coated on a meta-aramid textile substrate. The novelty of this work lies in the integrated design and evaluation of a CNT-modified polymeric PTC system in which composition-dependent conductive-network formation is directly linked to self-regulating electrothermal behavior, followed by its implementation on a flame-resistant meta-aramid textile for practical heating applications. The primary objective is to elucidate the relationship between CNT-induced conductive-network formation, temperature coefficient of resistance (TCR) behavior, and electrothermal self-regulation [32,33]. By systematically varying the PTC:CNT ink mass ratio from 95:5 to 80:20, the effects of the MWCNT-dispersed paste fraction on conductive-network formation, heating response, and thermal stability were investigated. The optimized polymer–CNT composite was further coated onto a meta-aramid textile substrate to fabricate a flexible surface-heating textile [34,35]. Its mechanical durability, electrical stability, and flame-retardant behavior were evaluated under repeated bending, washing, and thermal exposure conditions [36]. In addition, the optimized heater was assessed at the system level in an EV cabin-mimicking chamber against a commercial ceramic PTC heater, enabling the material-level electrothermal characteristics to be connected with practical heat-delivery behavior [37].

## 2. Materials and Methods

### 2.1. Preparation of Composite PTC Paste

In this study, a composite PTC paste with optimized electrical and rheological properties was formulated for textile coating. A commercial PTC carbon paste (PTC105, Seleniz, Republic of Korea) was blended with an MWCNT-dispersed carbon ink (Mcell, Republic of Korea) to prepare the hybrid PTC-CNT paste. According to the manufacturer’s safety data sheet, PTC105 contains carbon black (1–10 wt%), glass wool (20–30 wt%), and 1,1,3,3-tetramethylurea (50–60 wt%), while part of the detailed formulation remains proprietary. The MWCNT-dispersed paste consisted of 4 wt% MWCNT, 2 wt% ethyl cellulose, 80 wt% terpineol, 12 wt% silicone resin (RSN-0806), and 2 wt% dispersant (DISPERBYK 192).

The composite PTC-CNT pastes were prepared by blending the commercial PTC105 ink with the MWCNT-dispersed carbon ink at PTC105 ink–MWCNT-dispersed ink weight ratios of 95:5, 90:10, 85:15, and 80:20. This composition range was selected to systematically investigate the effects of the MWCNT-dispersed ink fraction on conductive-network formation, TCR behavior, PTC response sensitivity, and electrothermal stability. For clarity, the formulations are denoted by the PTC:CNT ink mass ratio. Because the MWCNT-dispersed paste contained 4 wt% MWCNT, PTC:CNT ink ratios of 95:5, 90:10, 85:15, and 80:20 correspond to nominal MWCNT content of 0.20, 0.40, 0.60, and 0.80 wt%, respectively, on a wet-paste basis. Because the detailed solid content of the commercial PTC105 paste is proprietary, the exact dry-basis MWCNT fraction in the fully cured composite could not be determined.

Prior to blending, the MWCNT-dispersed ink was subjected to ultrasonication to disrupt agglomerates. The sonicated MWCNT-dispersed ink was then mixed with the PTC105 ink according to the designated PTC:CNT ink ratios. The resulting mixtures were processed by three-roll milling to improve dispersion uniformity and obtain macroscopically homogeneous composite pastes (Figure 1) [18]. The pastes exhibited no visible phase separation, indicating macroscopically homogeneous dispersion after the mixing and milling processes. Finally, each paste was passed five times through a three-roll mill (EXAKT 50, EXAKT Advanced Technologies GmbH, Norderstedt, Germany) at a roller speed of 150 rpm for 30 min with a total batch size of 20 g to apply high shear and improve CNT dispersion within the polymer-based PTC matrix [18,38].

The apparent viscosity of the prepared PTC-CNT pastes was measured using a digital viscometer (DVNext HB, AMETEK Brookfield, Middleboro, MA, USA) equipped with a CP-52 spindle. Measurements were performed at a rotational speed of 5 rpm, corresponding to a shear rate of 10 s^−1^, at room temperature and approximately 60 °C. Each measurement was repeated three times under the same conditions, and the results are reported as means ± standard deviation (*n* = 3).

### 2.2. Fabrication of PTC-CNT–SHT

To verify the practical applicability of the composite PTC-CNT paste, a meta-aramid-based surface-heating textile (SHT) was fabricated using a flexible textile substrate [18]. The textile substrate consisted of an aramid-based composite yarn incorporating Ag-coated conductive filaments (diameter: 0.05 mm, line resistance: 4.18 Ω/m, Mcell, Seongnam, Republic of Korea). The fabric was woven into a plain-weave structure by L&T Materials Co., Ltd. (Gongju, Republic of Korea). Within an area of 750 × 750 mm^2^, ten conductive yarns were arranged in both the warp and weft directions at a spacing of 17 mm, and the gap between adjacent conductive yarn rows was maintained at 47 mm (Figure 2).

The composite PTC-CNT paste was deposited onto the textile substrate using a tape-casting method to achieve a uniform coating layer. A target coating thickness of 25 μm was selected for fabrication of the optimized PTC-CNT–SHT [39]. After coating, the samples were thermally cured at 130 °C for 15 min to remove the solvent completely and to stabilize the structure of the composite layer. The nominal coating thickness was set to 25 μm during deposition. After curing, cross-sectional SEM images confirmed the formation of a continuous PTC-CNT coating layer with an actual thickness of approximately 25 μm. The Ag-coated conductive filaments served as low-resistance current-distribution pathways across the coated textile surface.

### 2.3. Physical and Thermal Characterization of PTC-CNT–SHT

The electrical stability and mechanical durability of the textile-based surface heater coated with the composite PTC-CNT paste were quantitatively evaluated. For thickness-dependent durability evaluation, additional specimens with nominal coating thicknesses of 30, 60, 90, and 120 μm were prepared separately from the optimized 25 μm sample. For each thickness condition, samples were measured at three positions to obtain average values. The sheet resistance was measured using a four-point probe system (KeithLink Technology Co., Ltd., New Taipei City, Taiwan) following ASTM F1896 [40]. All samples were fabricated under identical coating and curing conditions (130 °C, 15 min), and the measurement environment was maintained at 25 °C and 45% relative humidity. The coating thickness was measured using a contact-type thickness gauge (547-401, Mitutoyo Corp., Kawasaki, Japan). The cross-sectional morphology of the coated layer was examined by scanning electron microscopy (S-4300, Hitachi, Tokyo, Japan).

The thermal stability of the PTC-CNT-coated m-aramid textiles was evaluated by thermogravimetric analysis (TGA, N-1500, SCINCO Co., Ltd., Seoul, Republic of Korea). The measurements were performed from room temperature to 600 °C at a heating rate of 10 °C min^−1^ under an Ar atmosphere. Samples with PTC:CNT ink ratios of 100:0, 95:5, 90:10, 85:15, and 80:20 were analyzed to compare the thermal stability of the coated textile system over and beyond the practical operating-temperature range.

Mechanical stability was assessed by a cyclic bending test. Because aramid-based fabrics exhibit high stiffness and low elongation, making them more sensitive to bending than tensile loading, rectangular specimens with dimensions of 2 cm (width) × 7 cm (length) were cut, and the bending direction was oriented perpendicularly to the principal yarn direction (Figure S1). The specimens were bent such that they were folded approximately in half along their length. The number of bending cycles was set to 1000, 2000, 3000, 4000, and 5000. After each cycle interval, the electrical resistance of the specimen was measured, and the relative resistance change, ΔR/R_0_, was calculated with respect to the initial resistance R_0_ to quantify the effect of bending on electrical properties [18].

Washability was evaluated using a conventional household washing machine under fixed washing conditions: water temperature of 25 °C, washing time of 74 min, detergent concentration of 1.0 g/L, and up to ten washing cycles. The surface resistance of the samples was measured before and after washing to assess the retention of electrical performance. Flame retardancy was examined using 100 mm × 55 mm specimens prepared from m-aramid-based PTC-CNT–SHT and Dewspo fabric-based PTC-CNT samples. The specimens were connected to a DC power supply and temperature sensors, and an increasing voltage was applied according to a stepwise protocol (Figure S2). The applied voltage was increased in steps of 5 V every 30 s up to a maximum of 30 V. To observe the ignition behavior directly, a match flame was brought into contact with the fabric surface, and changes in appearance and charring were compared before and after the test.

### 2.4. Electrical and Electrothermal Properties of PTC-CNT–SHT

The surface resistivity of the coated PTC-CNT–SHT specimens was measured in accordance with ASTM D257. Test specimens were prepared by coating a 50 × 50 mm effective area on a 60 × 60 mm m-aramid substrate. The Ag-coated conductive filaments were connected to the external DC power supply using alligator clips during electrothermal testing. All samples were conditioned for at least 24 h at 23 ± 2 °C and 50 ± 5% relative humidity prior to measurement to minimize the influence of ambient variations.

A guarded concentric-ring electrode assembly was used for the measurements, with an initial applied voltage of 10 V. After the current reached a steady state, the voltage was increased stepwise to 100 V and 500 V, and the corresponding current responses were recorded. The surface resistivity (Ω/sq) was calculated from the measured current and applied voltage using the geometric factors specified in ASTM D257 [41]. For each specimen, five positions were measured and the average value was used [42].

Heating performance was evaluated using a DC power supply at voltages of 12, 24, 36, 48, and 60 V. A constant voltage was applied for 1 h under each condition, while the heating area was fixed at 13.5 cm^2^ to ensure consistent comparison of power consumption per unit area. Resistance before and after voltage application was recorded using a digital multimeter. Instantaneous power consumption was calculated using Ohm’s law [43] and then normalized by the heating area to obtain the power density (W/m^2^).

(1) $$P=\frac{V^{2}}{R}$$

For the composition-dependent comparison of the PTC-CNT–SHT specimens at 24 V, an active heating area of 18 cm^2^ was used. The spatial temperature distribution of the optimized 90:10 PTC-CNT–SHT was evaluated using a non-contact infrared thermal imaging camera (FLIR, MSX Technology, Wilsonville, OR, USA). The heater was operated at a constant voltage of 24 V, and thermal images were acquired after 1 min and 1 h of operation.

### 2.5. Thermal Behavior in Cabin-Mimicking Chamber

To evaluate the applicability of the m-aramid-based PTC-CNT–SHT for EV cabin heating, a dedicated chamber was constructed to simulate the thermal environment of a passenger compartment (Figure 3). The internal dimensions of the chamber were 1520 mm × 1000 mm × 1220 mm, and the outer walls consisted of thermal insulation, rigid panels, and a transparent observation window. Unless otherwise specified for the fan-placement experiments, the chamber tests were conducted under natural convection. An integrated mounting bracket was installed on the front to provide reproducible mounting conditions for the top-mounted PTC-CNT–SHT configuration. The initial internal temperature was set within 18–22 °C, and the difference in initial temperature between experiments was controlled to be less than 0.5 °C. The ambient laboratory temperature was maintained at 20 ± 1 °C. During the experiments, the chamber door remained closed and external air inflow was blocked to prevent disturbances in the thermal balance. Temperature was measured using eight K-type thermocouples (CH01–CH08). CH01 was placed at the upper center of the chamber to represent the perceived temperature near the upper body and face of a seated passenger [44,45]. CH02–CH06 were attached at five equally spaced positions along the mid-height plane in front of the heater to evaluate lateral heat spreading and temperature uniformity of the surface heater. CH07 and CH08 were located at the lower corners to represent “dead zones” where warm air typically reaches last in an actual vehicle interior. This arrangement of upper, middle, and lower sensors was designed to quantify the vertical temperature gradient (thermal stratification) and lower-zone cooling effects that are critical in EV cabin heating: CH01 was used to evaluate the upper-zone air-temperature response, CH02–CH06 were used to assess lateral temperature distribution at the mid-height plane, and CH07–CH08 were used to characterize the lower-zone thermal response. The thermocouples had a measurement accuracy of ±0.1 °C, and the temperature data were recorded at 1 min intervals. Prior to each experiment, the initial readings of all thermocouples were adjusted to within 0.5 °C of each other to eliminate sensor offsets [44].

A programmable DC power supply (RD6006W, Hangzhou Ruideng Technology Co., Ltd., Hangzhou, China) was used to drive the heaters and was controlled using Riden Power Supply software (version 1.0.0.8). For each specimen, the initial resistance was measured, and the supply voltage corresponding to the target power levels (100, 200, 230 and 240 W) was calculated and set. The output stability of the power supply was maintained within ±0.5%. The 100 W condition represents an “eco-heating” or low-power maintenance mode in EVs, while 200 W corresponds to a typical comfort-heating level [44]. The 230 W condition mimicked a high-power boost mode immediately after heater activation, and the 240 W condition was chosen to clearly distinguish the effects of fan placement on convective flow and heat distribution by providing a sufficiently strong heating input. Voltage and current were measured at 1 s intervals using a data logger, and the instantaneous power was calculated as P(t)=V(t)I(t). One-minute averages were stored for subsequent analysis. The total energy consumption (Wh) was obtained by time integration of the power profile over the experimental duration [46]. All temperature, voltage, and current data were acquired synchronously using a multichannel data logger and backed up as raw data immediately after each experiment.

Three types of heaters were tested: PTC-CNT–SHT top, PTC-CNT–SHT bottom, and a reference BaTiO_3_-based commercial ceramic PTC heater (hereafter commercial PTC). The PTC-CNT–SHT top was mounted on the upper front frame of the chamber so that the heating surface was directly exposed to the cabin air, mimicking an upper front-panel heater in an EV. The PTC-CNT–SHT bottom sample was installed in intimate contact with the floor surface, simulating a floor or footwell heater with direct heat conduction into the floor structure and associated cooling effects. The active heating area of both PTC-CNT–SHT samples was 1000 mm × 1110 mm, and the electrode layout was kept identical to ensure reproducible current paths and power-input conditions. The commercial PTC heater module, comprising a BaTiO_3_ ceramic PTC element integrated with a built-in fan, was mounted near the top of the chamber and operated under airflow conditions similar to those in automakers’ EV HVAC systems.

Air-circulation conditions were evaluated using the PTC-CNT–SHT top. An auxiliary fan providing an airflow comparable to that of a typical passenger-car HVAC system was employed. The fan power consumption (3.8 Wh/h) was included in the total energy consumption (Wh) when analyzing the commercial PTC heater. The fan could be positioned at the front, rear, or of the top-mounted PTC-CNT–SHT to realize different convective patterns: vertical forced convection (front), rear recirculating convection (rear), and side-to-top mixed convection (side). In experiments without the fan, forced convection was eliminated and only natural convection was allowed to evaluate the intrinsic surface-heating behavior of the PTC-CNT–SHT.

The same experimental procedure was applied to all power conditions. After cleaning the chamber and initializing the instruments, the contact between the specimens and sensors was checked. Each specimen was mounted and allowed to equilibrate for 10 min under no power to approach thermal equilibrium between the chamber and the heater. Subsequently, the voltage corresponding to the target power level (100, 200, 230, or 240 W) was applied to start the experimental run [44]. The 100 and 200 W conditions were maintained for 300 min, the 230 W condition for 120 min, and the fan-placement experiments at 240 W for 60 min. After each run, the power was switched off and the system was allowed to cool naturally for 10 min. During all experiments, any actions that could disturb the heat balance, such as opening the chamber door or changing cable positions, were strictly prohibited to ensure reproducibility between runs [44].

## 3. Results and Discussion

### 3.1. Rheological and Morphological Characteristics of the PTC-CNT System

In this study, “self-regulating behavior” referred to a self-limiting electrothermal response under constant-voltage operation in which the temperature rise was moderated by the temperature-dependent resistance increase of the PTC-CNT composite. The apparent viscosity of the PTC-CNT pastes was measured at a fixed shear rate of 10 s^−1^ to evaluate the effects of formulation ratio and temperature on the flow characteristics of the coating paste. At room temperature, the apparent viscosity generally decreased with increasing fraction of the MWCNT-dispersed paste, from 21,950 cP for the 100:0 formulation to 11,520 cP for the 80:20 formulation. All formulations exhibited substantially lower viscosity at 60 °C, indicating a pronounced temperature dependence of the paste flow behavior. Although the 90:10 formulation showed a slight deviation from the overall composition-dependent trend at 60 °C, the results demonstrate that the PTC:CNT ink ratio affects not only the electrical characteristics but also the processing characteristics of the hybrid paste. The measured viscosity range was compatible with the coating process employed in this study, enabling the formation of continuous PTC-CNT layers on the m-aramid substrate. These results indicate that control of the formulation ratio is important for maintaining suitable processability during coating, as well as for tuning the electrothermal properties of the resulting heater (Figure 4).

Figure 5 presents the cross-sectional morphology of the PTC-CNT–SHT textile heater. The bare m-aramid fabric exhibited a porous woven structure without any surface coating (Figure 5a). After deposition of the composite PTC-CNT paste, a continuous coating layer with an actual thickness of approximately 25 μm was formed on the textile surface (Figure 5b). The coating layer remained closely associated with the fibrous substrate without noticeable delamination or large interfacial voids.

To further examine the morphology of the optimized composition, the surface and microstructure of the 90:10 PTC-CNT coating were observed at higher magnification (Figure 6). Compared with the relatively smooth surface of the bare m-aramid fibers (Figure 6a), the coated fibers exhibited a heterogeneous surface morphology (Figure 6b). Higher-magnification images of the cured coating (Figure 6c,d) revealed a continuous matrix containing dispersed rounded particulate features and finer surface structures. Because the commercial PTC paste contains multiple constituents and part of its detailed formulation is proprietary, the individual particulate phases observed in the SEM images were not specifically assigned.

The thermal stability of the PTC-CNT-coated m-aramid textiles was subsequently evaluated by thermogravimetric analysis (Figure 7). All compositions exhibited similar overall mass-loss behavior, with only minor weight changes within the practical operating-temperature range of the heater. At the target operating temperature of approximately 130 °C, all coated textile samples retained 97.9–98.7% of their initial mass, and no additional pronounced mass loss was observed up to 150 °C. More substantial mass loss occurred only at higher temperatures, particularly above approximately 250–300 °C. Because the TGA measurements were performed on the complete PTC-CNT-coated m-aramid structure, the observed mass-loss profiles represent the combined thermal response of the coating and textile substrate rather than the decomposition behavior of individual paste constituents.

### 3.2. Electrical and Electrothermal Characteristics of the PTC-CNT Composite

Although individual MWCNTs could not be directly resolved in the SEM images, the systematic decrease in sheet resistance with increasing MWCNT-dispersed ink fraction indicates the development of increasingly effective conductive pathways within the composite coating. These composition-dependent changes in electrical connectivity are closely related to the electrothermal and self-regulating behavior discussed below [11,47].

MWCNTs are well known as conductive carbon materials with low electrical resistance and a negative temperature coefficient (NTC) [48]. When they are incorporated into a PTC ink, they provide additional conductive pathways that reduce the initial resistance and modify the temperature-dependent electrical response of the composite. The surface resistance of the pure PTC105 ink was measured to be approximately 1515 Ω/sq, and both the power consumption and the equilibrium temperature increased as the applied voltage was raised. However, the temperature rise became saturated around 85 °C, and at higher voltages the resistance increased sharply. At 60 V, this resistance increase was sufficiently large to reduce the power consumption despite the higher applied voltage (Figure 8a, Table 1). This behavior reflects the intrinsic self-regulation of PTC materials, in which resistance increases steeply with temperature. At the same time, it implies that the pure PTC105 ink alone is not sufficient to reach the target temperature of ~130 °C required for automotive heater applications.

To quantify this behavior, the temperature coefficient of resistance (TCR), α(T), was introduced as a measure of the resistance variation with temperature. TCR is defined as in Equation (2), representing the sensitivity of resistance to temperature changes [19,28,49,50]:

(2) $$\begin{array}{c} \alpha (T)=\frac{1}{R(T)}\frac{dR}{dT} \end{array}$$

The average TCR, α_avg_, was therefore calculated from the initial and final resistance values over the practical heating range using Equation (3) [51,52]:

(3) $$\alpha =\frac{R_{2}-R_{1}}{R_{1}(T_{2}-T_{1})}$$

where $T_{1}$ and $T_{2}$ are the initial and final temperatures, and $R_{1}$ and $R_{2}$ are the corresponding resistances. TCR is a key parameter for evaluating both the thermal stability and the self-regulation performance of a heater [53]. If the TCR is excessively large, the temperature response is fast, but the resistance increases too steeply, making it difficult to reach the desired operating temperature. Conversely, if the TCR becomes too small due to an excessively dense conductive network, the self-regulation effect weakens, which may increase the risk of overheating or thermal runaway.

For the pure PTC105 paste, the average TCR at 24 V was relatively low, which helped to suppress power consumption, but also prevented the system from reaching the target temperature of ~130 °C. As summarized in Figure 8b, the voltage-dependent changes in power consumption, average TCR, power density, and energy consumption per degree of temperature rise further illustrate the trade-off between heat generation and self-regulating behavior in the pure PTC105 system. Therefore, for automotive heater applications, it is necessary to tailor the composition via CNT incorporation in such a way that the initial resistance and heating rate are optimized while maintaining an appropriate TCR.

Accordingly, composite pastes were prepared by blending PTC105 with the MWCNT-dispersed paste at PTC:CNT ink mass ratios of 95:5, 90:10, 85:15, and 80:20, and their heating characteristics and electrical responses were compared (Table 2). When the PTC:CNT ratios were 100:0, 95:5, 90:10, 85:15, and 80:20, the initial sheet resistances were 1500, 1400, 1100, 1000, and 800 Ω/sq, respectively, showing a gradual decrease with increasing CNT content. As the fraction of the MWCNT-dispersed paste increased, the initial resistance decreased, the heating rate accelerated, and the attainable temperature increased. The 95:5 composition maintained relatively stable heating around 115 °C, but still fell short of the desired 130 °C. In contrast, the 90:10 composition exhibited a moderate resistance change of about 174 Ω while reaching a maximum temperature of 130.2 °C, thereby achieving both electrothermal performance and self-regulation stability.

Among the CNT-containing composite formulations, the average TCR decreased from 3.196% K^−1^ for the 95:5 composition to 1.291% K^−1^ and 0.698% K^−1^ for the 90:10 and 85:15 compositions, respectively (Table 2). This trend suggests that increasing CNT loading established more persistent conductive pathways within the PTC matrix, thereby moderating the resistance increase during heating. The 95:5 composition retained a relatively strong PTC response, but reached only approximately 117 °C after 1 h, which remained below the target operating temperature. In contrast, the 85:15 composition exhibited a lower average TCR and exceeded the desired temperature range, reaching approximately 148 °C. The 90:10 formulation showed an intermediate average TCR response and reached approximately 130 °C after 1 h. It was therefore selected as the optimized composition for subsequent fabrication of the PTC-CNT–SHT.

To further evaluate the spatial heating behavior of the optimized 90:10 PTC-CNT–SHT, infrared thermal imaging was performed under a constant applied voltage of 24 V. After 1 min of operation, the measured spot temperature reached 95.9 °C, and after 1 h it had increased to 130.8 °C (Figure 9), in good agreement with the corresponding time-dependent heating measurements. The infrared images confirmed that the elevated-temperature region extended across the active coated area rather than being confined to a single localized point. Although some spatial temperature variation was observed, no severe localized thermal runaway was evident during the 1 h operation.

At the highest MWCNT-dispersed paste fraction, the 80:20 composition reached approximately 250 °C. Because of this excessive temperature rise, reliable resistance-related parameters could not be obtained under the present test conditions, further indicating that excessive conductive-network formation compromises the desired self-regulating behavior.

Consequently, under the 24 V condition, the 90:10 PTC:CNT ink composition exhibited the most balanced performance in terms of heating rate, power consumption, target-temperature attainment, and self-regulation stability. This composition was therefore selected as the optimal formulation for subsequent design of the PTC-CNT–SHT for EV cabin-heating applications. The optimized 90:10 composition exhibited a balanced electrothermal response, where sufficient conductive pathways were formed while maintaining stable PTC-driven self-regulation. At higher CNT content, excessive conductive-network formation reduced the temperature-dependent resistance variation, thereby weakening the self-regulating behavior.

Figure 10 provides a schematic summary of the CNT-loading-dependent electrothermal behavior of the PTC-CNT composite ink and its integration into the m-aramid textile heater. At lower CNT content, the limited number of conductive pathways restricts joule heating and prevents the target temperature from being reached. In contrast, the optimized PTC ratio of 90:10 provides sufficient electrical conduction while retaining the temperature-dependent resistance increase required for stable heating near 130 °C. At higher CNT content, the increased density of CNT-mediated pathways reduces the resistance modulation during heating and promotes excessive temperature rise. Based on this balance, the 90:10 composition was selected for subsequent fabrication and evaluation of the PTC-CNT–SHT.

Choi et al. previously demonstrated a PTC-MWCNT-based surface-heating textile for wearable heating applications, providing an earlier example of self-regulating textile heating [18]. In comparison, the present study extends the concept toward higher-temperature operation near 130 °C and EV-oriented chamber-scale heat-delivery evaluation. Jia et al. reported that a c-MWCNT–aramid nanofiber film containing 50 wt% c-MWCNT reached a surface temperature above 180 °C at 10 V [16], while Wang et al. reported an aramid nanofiber–CNT-coated electrothermal paper that reached a saturated temperature of approximately 190 °C at 25 V [19]. These CNT–aramid systems demonstrate the high electrothermal performance attainable from highly conductive carbon networks; however, they primarily function as conventional resistive joule heaters. In contrast, the present PTC-CNT–SHT was designed to balance sufficient electrical conduction with a temperature-dependent resistance increase, and the optimized 90:10 composition stabilized near 130 °C at 24 V. Thus, the objective of the present system is not to maximize the attainable temperature, but to achieve controlled high-temperature heating with PTC-mediated self-regulating behavior for EV-oriented surface-heating applications.

### 3.3. Mechanical Durability and Flame-Retardant Behavior

In practical use, PTC-CNT–SHT is subjected to various mechanical loads such as repeated compression due to seating, vibration during vehicle operation, and bending or stretching associated with curved surfaces. The mechanical robustness of the heater under such conditions is directly linked to its electrical reliability. For this reason, both cyclic bending tests and washing durability tests were conducted to evaluate the durability of the composite PTC-CNT–SHT [54].

Rectangular specimens (2 cm × 7 cm) were prepared, and PTC-CNT composite layers with thicknesses of 30, 60, 90, and 120 μm were coated on meta-aramid (M) and Dewspo (D) substrates. Cyclic bending tests were carried out up to 5000 cycles, with the resistance measured after each 1000-cycle interval [55]. For both the m-aramid and Dewspo fabrics, thin coatings of 60 μm or less (M30, M60, D30, D60) showed relatively small resistance changes, with resistance changes of less than 1% even after 5000 bending cycles. In contrast, thicker coatings of 90 μm or more (M90, M120, D90, D120) exhibited a gradual increase in resistance with increasing cycle number. For the thickest 120 μm coatings, the resistance increased by approximately 40–50% after 5000 cycles, indicating that excessive coating thickness is a major factor contributing to reduced mechanical durability under bending stress (Figure 11a).

Washing durability tests showed a similar trend. Most specimens maintained their initial resistance with negligible variation up to five washing cycles, but a pronounced resistance increase was observed in the thick coatings after ten cycles [56]. Notably, the Dewspo specimen with a 120 μm coating exhibited more than a twofold increase in resistance after ten washing cycles, demonstrating its relative vulnerability to moisture and mechanical abrasion during washing. In contrast, the 30 μm coatings retained their resistance within a 5% variation, indicating stable electrical behavior under repeated washing (Figure 11b). These results indicate that a coating thickness of ~30 μm provides a favorable balance between mechanical flexibility and electrical reliability, maintaining stable performance under both cyclic bending and washing. This behavior was consistent for both m-aramid and Dewspo substrates, suggesting that the 30 μm PTC-CNT layer can provide sufficient durability and reliability for seat and interior applications in vehicles [12].

Flame retardancy is another critical property for safe application of heating textiles. In this study, flame-retardant behavior was assessed by monitoring the thermal response under applied voltage and the reaction to direct flame contact. Under the present applied-voltage test, no visible flame or ignition was observed from the PTC-CNT coating. This result suggests that the ignition behavior of the heater was governed primarily by the textile substrate rather than by the coating layer under the tested conditions. However, the Dewspo-based specimens began to deform near 150 °C, and visible combustion occurred at 188 °C, with flame formation at approximately 193 °C (Figure S3a–c).

In contrast, the m-aramid-based specimens began to emit smoke at around 212 °C, and even under 30 V applied voltage, only structural deformation was observed at approximately 248 °C without ignition or charring (Figure S3d–f). This indicates that the m-aramid substrate remains structurally stable at temperatures more than 60 °C higher than those tolerated by the Dewspo fabric.

Direct flame-contact tests further highlighted this difference. Dewspo specimens coated with the PTC-CNT composite ink exhibited immediate burning upon flame contact, leading to severe damage and sustained combustion, as shown in Figure 12b. In contrast, specimens based on m-aramid coated with either PTC or PTC-CNT composite ink extinguished the flame spontaneously within approximately 20 s after flame removal and retained their original shape without noticeable visual damage (Figure 12d).

These results indicate that the m-aramid substrate provides effective suppression of ignition not only under high-temperature operation with applied voltage, but also under direct flame exposure [57]. In combination with the PTC-CNT composite ink, it affords both structural stability and flame safety. While Dewspo fabric offers advantages in processability and low weight, it shows clear limitations in flame retardancy. The m-aramid-based composite, on the other hand, can satisfy stringent flame-retardant requirements for high-temperature and high-risk environments [58]. Consequently, a structure in which the PTC-CNT composite ink is coated on an m-aramid fabric is considered a more suitable configuration when heater safety is a primary design constraint. Such composite architecture is a promising candidate for heating elements in EV seats, aircraft interiors, and industrial heating materials that must meet rigorous flammability standards.

### 3.4. Electrothermal Performance Evaluation in a Cabin-Mimicking Chamber

To evaluate the practical electrothermal performance of the optimized 90:10 PTC-CNT–SHT under controlled chamber conditions, comparative thermal tests were carried out using top-mounted PTC-CNT–SHT, bottom-mounted PTC-CNT–SHT, and a BaTiO3-based commercial ceramic PTC heater. As performance metrics, the average temperature rise across the eight thermocouple channels (ΔT_avg_), the upper-center air-temperature rise at CH01 (ΔTCH01), the total electrical energy consumption, and the average input power were compared. Because the tested heaters differed in heat-transfer mode, mounting position, and active heating area, the chamber experiment was interpreted as a system-level comparison rather than as an intrinsic material-efficiency comparison. Figure 13 summarizes the results in terms of power-input level, spatial temperature distribution, and fan placement, while Table 3 presents representative chamber-heating results obtained under the nominal 200 W condition [5].

At 100 W and 200 W, the top-mounted PTC-CNT–SHT consistently exhibited the highest ΔT_avg_ among the three heaters (Figure 13a). Under the 100 W eco-heating condition, the ΔT_avg_ of the top sample was 8.6 °C, which is approximately 32% higher than that of the commercial PTC heater (6.6 °C). The bottom-mounted PTC-CNT–SHT sample (7.3 °C) also produced a 12% larger ΔT_avg_ than the commercial PTC heater. Under the 200 W comfort-heating condition, the ΔT_avg_ values of the top-mounted PTC-CNT–SHT, bottom-mounted PTC-CNT–SHT, and commercial PTC heater were 16.0, 11.0, and 13.0 °C, respectively, indicating that the top-mounted configuration maintained the highest average chamber-temperature rise at both power levels.

Under the nominal 200 W condition, the total energy consumption and average input power of the three heater configurations were comparable over the 300 min test period (Table 3). Under these matched system-level input conditions, the PTC-CNT–SHT top configuration produced the largest average chamber-temperature rise, reaching 16.0 °C compared with 13.0 °C for the commercial PTC heater. The PTC-CNT–SHT bottom configuration showed a lower ΔT_avg_ of 11.0 °C, which was consistent with heat transfer to the floor-contact structure [5,6].

The upper-center air-temperature rise at CH01 further illustrates the spatial heating response of the three heater configurations (Figure 13b). At 100 W, the ΔTCH01 values for the top-mounted and bottom-mounted PTC-CNT–SHT were 29.9 and 23.2 °C, respectively, compared with 18.8 °C for the commercial PTC heater. Under the 200 W condition, the corresponding ΔTCH01 values were 64.8, 40.5, and 28.6 °C for the top-mounted PTC-CNT–SHT, bottom-mounted PTC-CNT–SHT, and commercial PTC heater, respectively. At 230 W, the top-mounted PTC-CNT–SHT reached approximately 80.2 °C at CH01, whereas the commercial PTC heater reached approximately 48.3 °C. These results indicate that the top-mounted textile heater generated a stronger upper-zone air-temperature response under the present chamber-test conditions.

The three heaters also produced distinct vertical temperature profiles (Figure 13c). At 100 W, the PTC-CNT–SHT top configuration generated the largest upper-zone temperature rise, whereas the bottom configuration produced a relatively stronger lower-zone heating effect. The commercial PTC heater showed a more moderate vertical profile, but a lower overall temperature rise than the PTC-CNT–SHT top configuration. These results indicate that the thermal distribution within the chamber can be modulated by heater placement, suggesting the potential of the textile heater for localized and distributed heating applications.

The influence of fan placement on the convective pattern is summarized in Figure 13d. Direct front airflow reduced the chamber-temperature response, consistent with enhanced convective cooling at the heater surface. In contrast, side fan placement produced the most uniform temperature distribution among the upper, middle, and lower zones. These results suggest that airflow direction is an important design parameter for optimizing the heat-transfer performance of flexible polymer–CNT composite heaters. Because the heater configurations differed in active heating area, mounting position, and airflow condition, the chamber-heating results are discussed as system-level responses. Under the nominal 200 W condition, the top-mounted PTC-CNT–SHT produced the largest average chamber-temperature rise and upper-center air-temperature response among the tested configurations.

Overall, under the 100 and 200 W conditions, the top-mounted PTC-CNT–SHT consistently produced higher average chamber-temperature rises than the commercial PTC heater. It also exhibited a 59–127% higher upper-center air-temperature rise at CH01 over the examined operating conditions. The bottom-mounted PTC-CNT–SHT produced a lower average chamber-temperature rise, but showed a relatively stronger lower-zone heating response, suggesting its potential for lower-zone localized heating applications. Under the present fan-placement test, side airflow provided the most uniform temperature distribution among the upper, middle, and lower zones (Figure 13d) [6].

## 4. Conclusions

In this study, a self-regulating PTC-CNT–SHT was developed by controlling the PTC:CNT ink composition and integrating the optimized composite with a meta-aramid textile substrate. Among the investigated formulations, the 90:10 PTC:CNT ink ratio exhibited the most balanced electrothermal behavior, reaching approximately 130.2 °C after 1 h at 24 V while maintaining an average TCR of 0.01291 K^−1^. The optimized heater also showed stable electrical performance during repeated bending and washing, while the meta-aramid substrate provided improved resistance to thermal exposure and ignition.

Infrared thermal imaging confirmed that the elevated-temperature region extended across the active heating area without severe localized thermal runaway. In addition, the cabin-mimicking chamber test demonstrated the practical heat-delivery capability of the optimized PTC-CNT–SHT at the system level: under the nominal 200 W condition, the top-mounted configuration produced an average chamber-temperature rise of 16.0 °C.

These results demonstrate that controlling the CNT-mediated conductive network provides an effective strategy for balancing electrical conduction, temperature-dependent resistance, and self-regulating heating behavior in flexible textile heaters. The developed PTC-CNT–SHT therefore shows potential for spatially distributed EV cabin-heating applications. Future work should focus on long-term operational stability, optimization of electrode and heater architecture, and direct characterization of temperature-dependent conductive-network evolution to further clarify the underlying PTC mechanism.

## Figures and Tables

**Figure 1:**
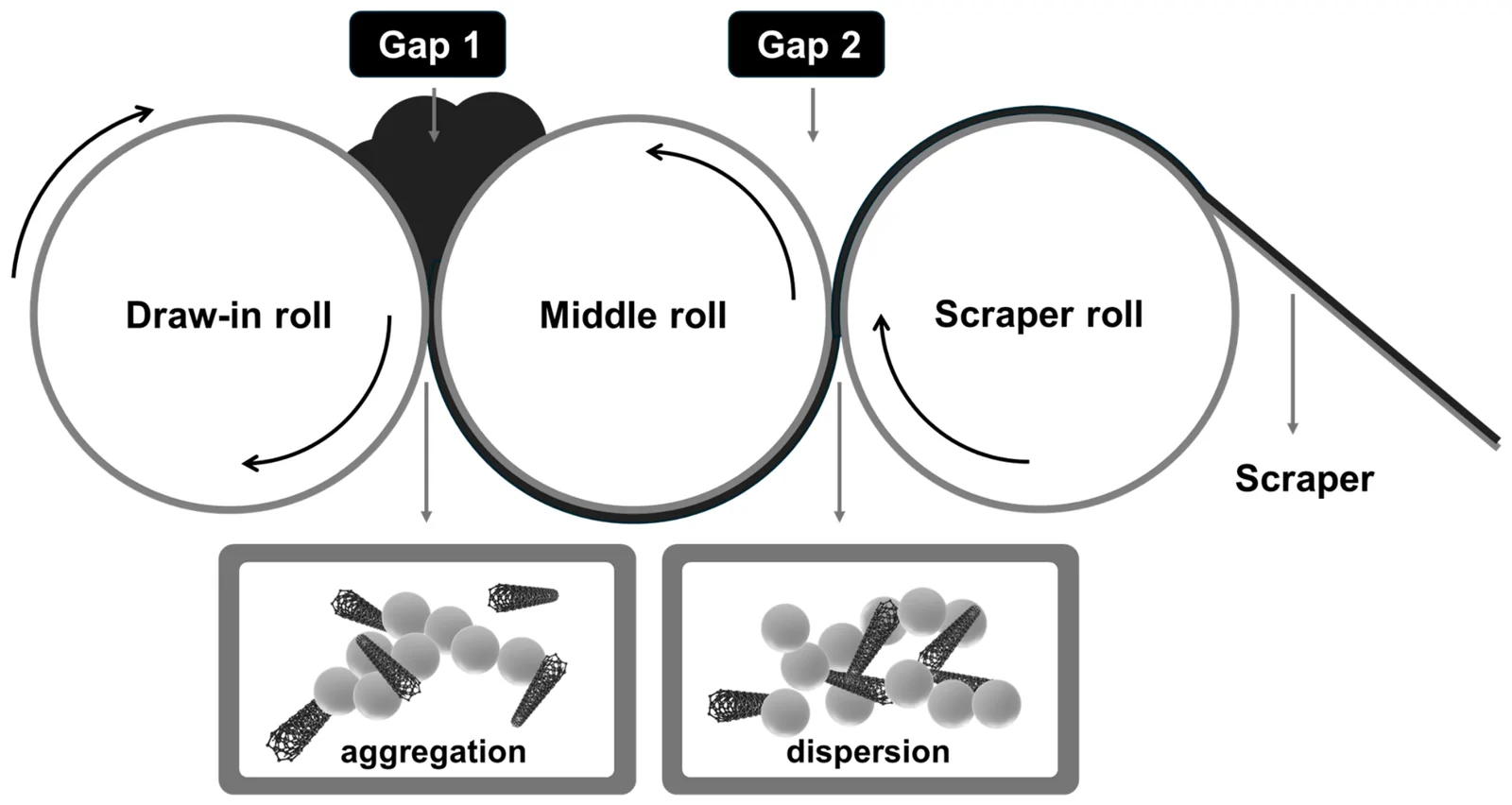
Preparation of uniformly dispersed PTC-CNT paste through repeated three-roll milling.

**Figure 2:**
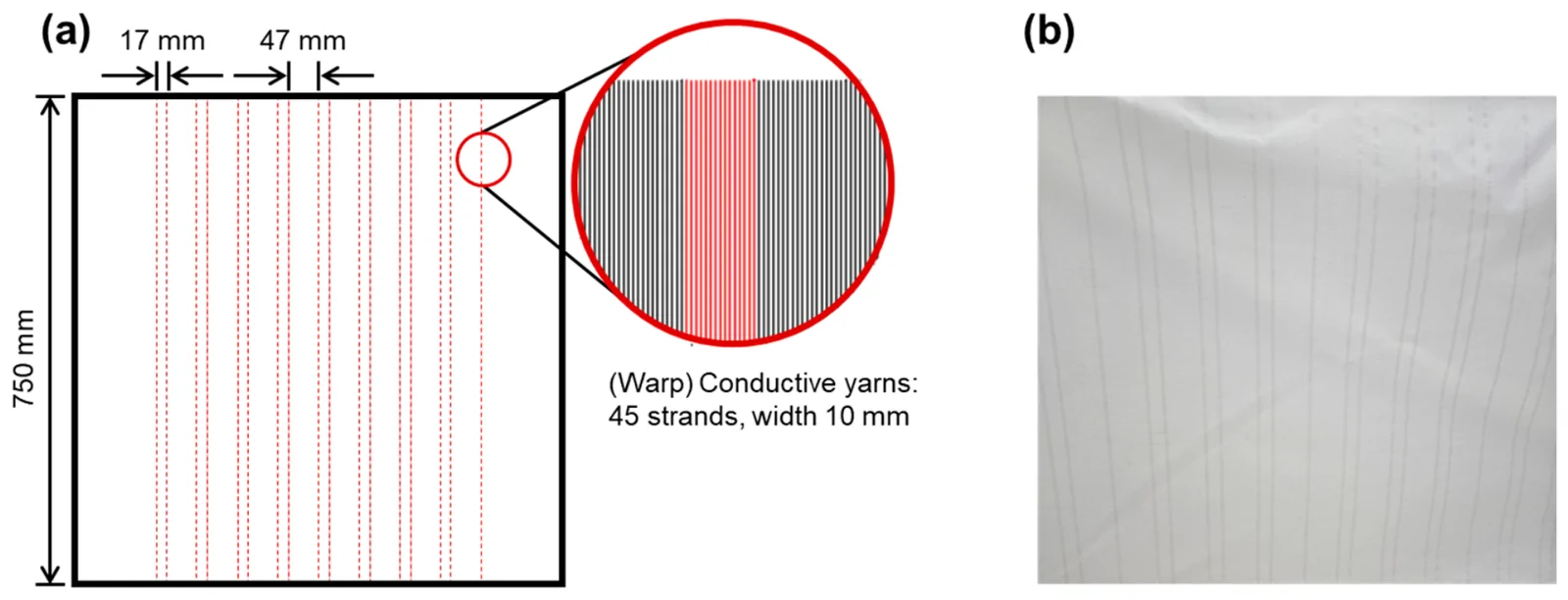
(**a**) Schematic illustration of the conductive embroidery on m-aramid fabric. (**b**) Photograph of the actual embroidered sample.

**Figure 3:**
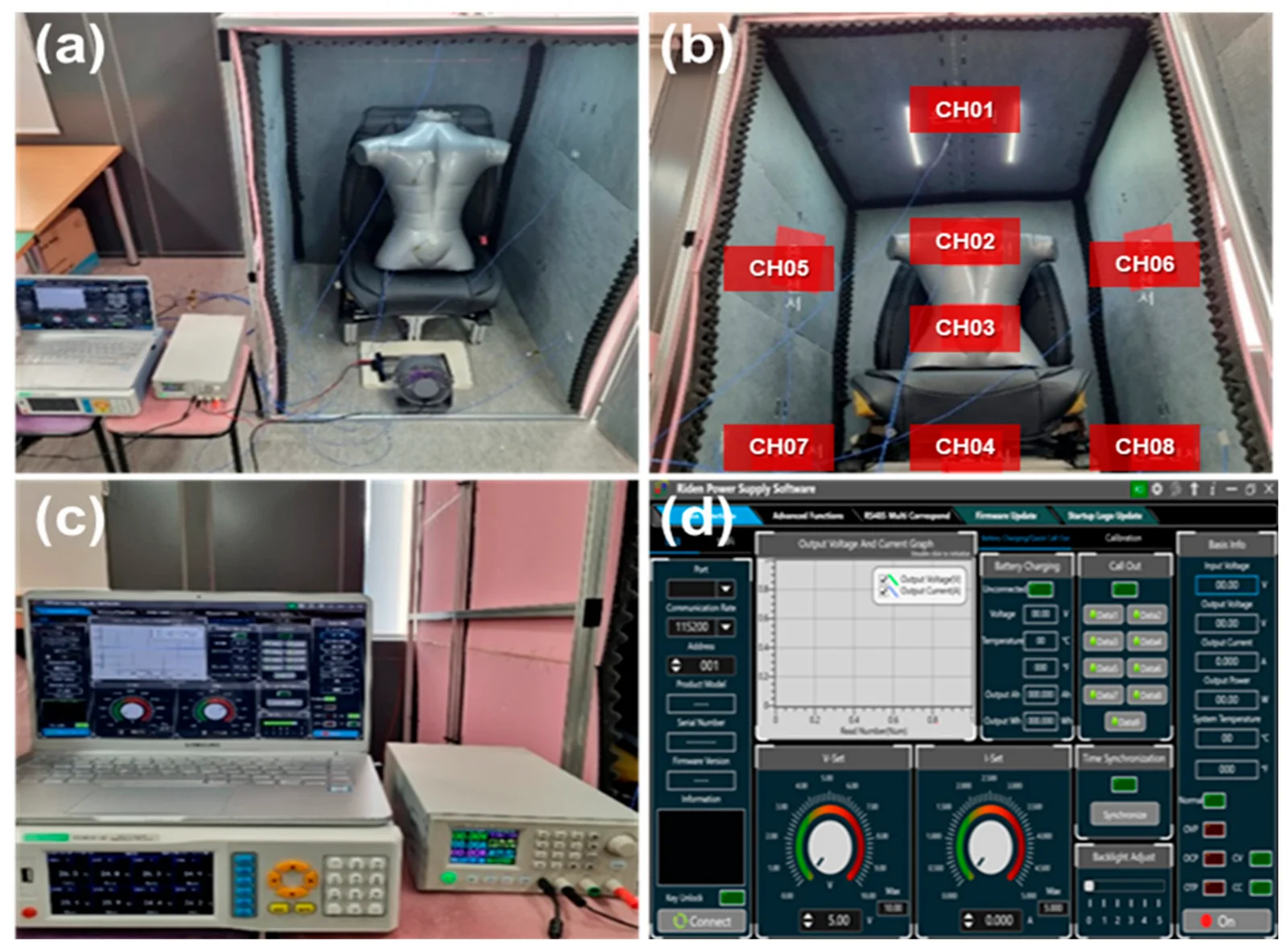
EV cabin-mimicking chamber and measurement system for evaluating the heating performance of PTC-CNT–SHT. (**a**) Overall view of the EV cabin thermal behavior simulation chamber equipped with a front mounting frame for m-aramid-based PTC-CNT–SHT samples, (**b**) positions of the K-type thermocouples (CH01–CH08) distributed in the upper, middle, and lower regions of the chamber, (**c**) DC power supply and multichannel data logger used for power delivery and signal acquisition, (**d**) control and data acquisition software for real-time monitoring and recording of voltage, current, and temperature.

**Figure 4:**
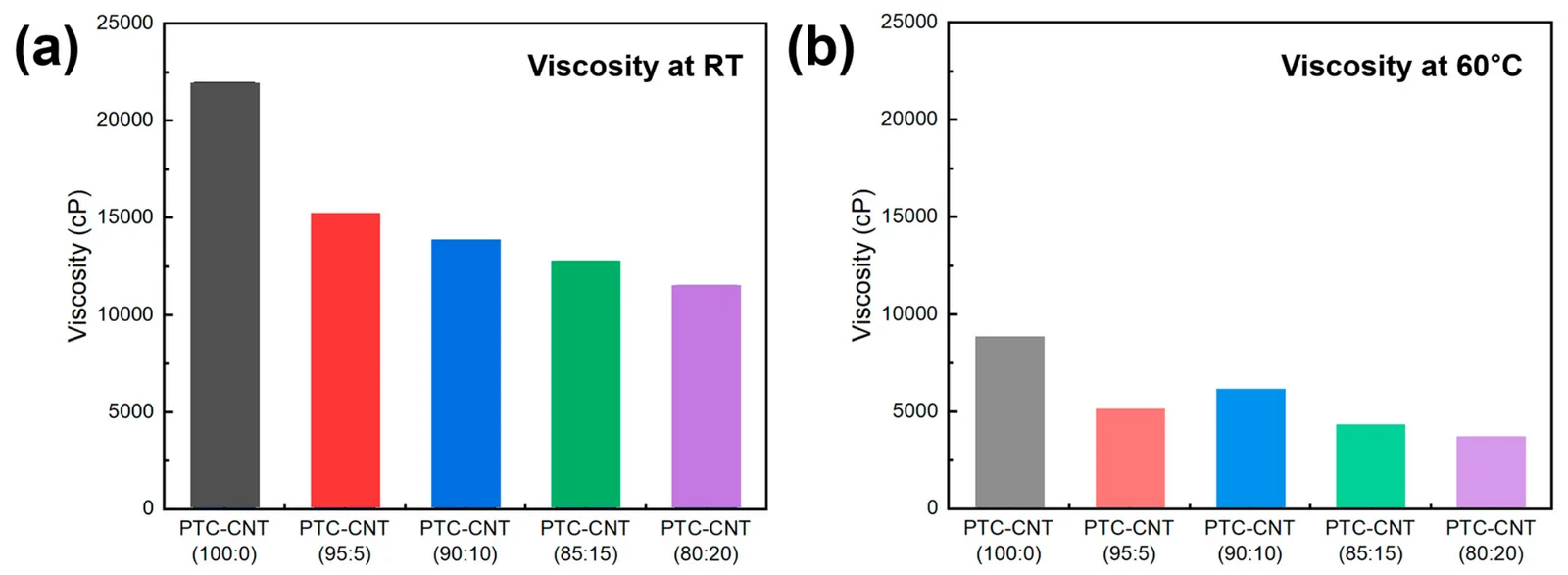
Apparent viscosity of PTC-CNT pastes with different PTC:CNT ink ratios measured at (**a**) room temperature and (**b**) 60 °C at a fixed shear rate of 10 s^−1^.

**Figure 5:**
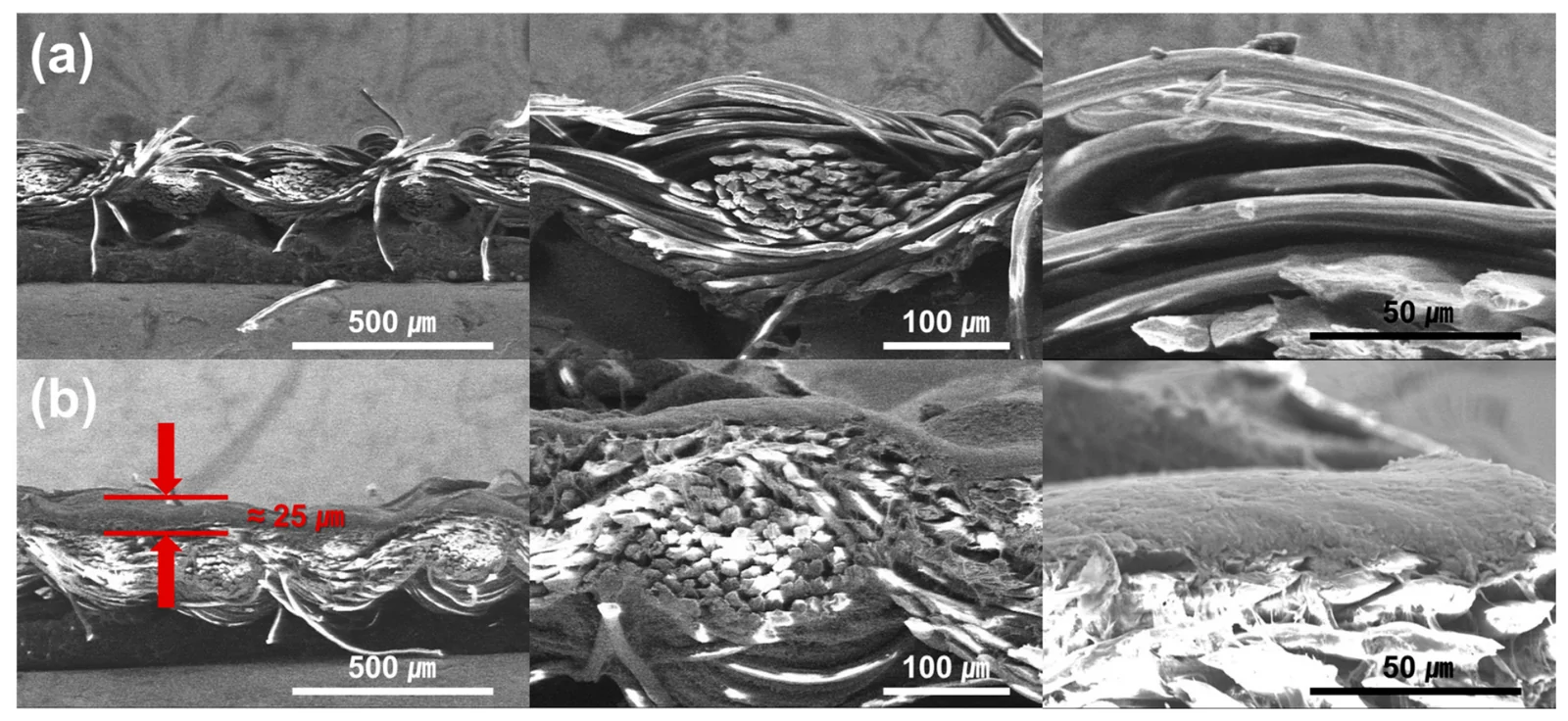
Cross-sectional SEM images of the m-aramid substrate. (**a**) Bare m-aramid fabric without coating. (**b**) PTC-CNT–SHT sample showing the coated PTC-CNT layer with a thickness of approximately 25 μm. The conductive-network formation is inferred from the composition-dependent electrical and electrothermal behavior.

**Figure 6:**
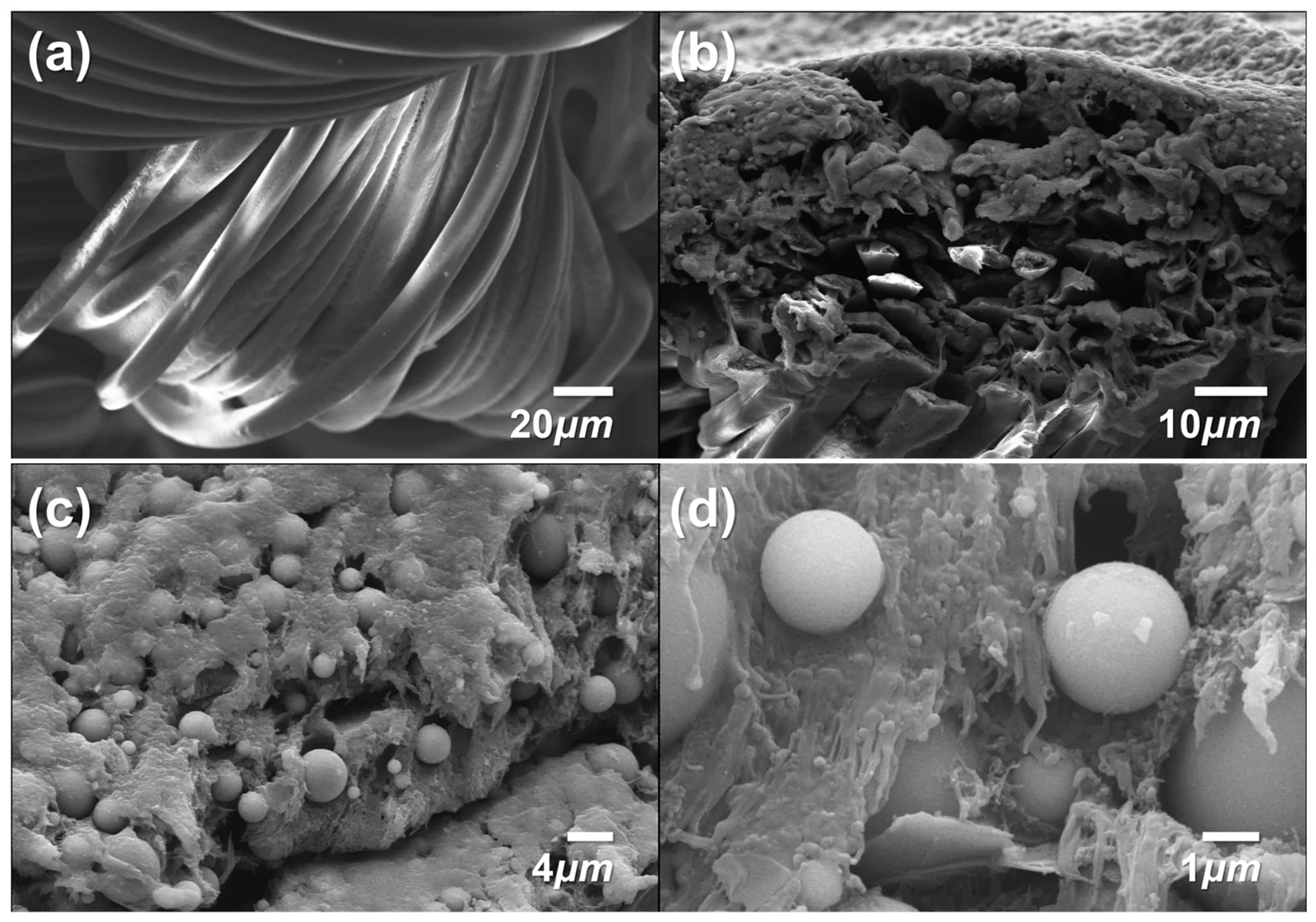
SEM images of the bare m-aramid fibers and the optimized 90:10 PTC-CNT coating. (**a**) Bare m-aramid fibers, (**b**) coated fibers, and (**c**,**d**) higher-magnification images of the cured PTC-CNT coating.

**Figure 7:**
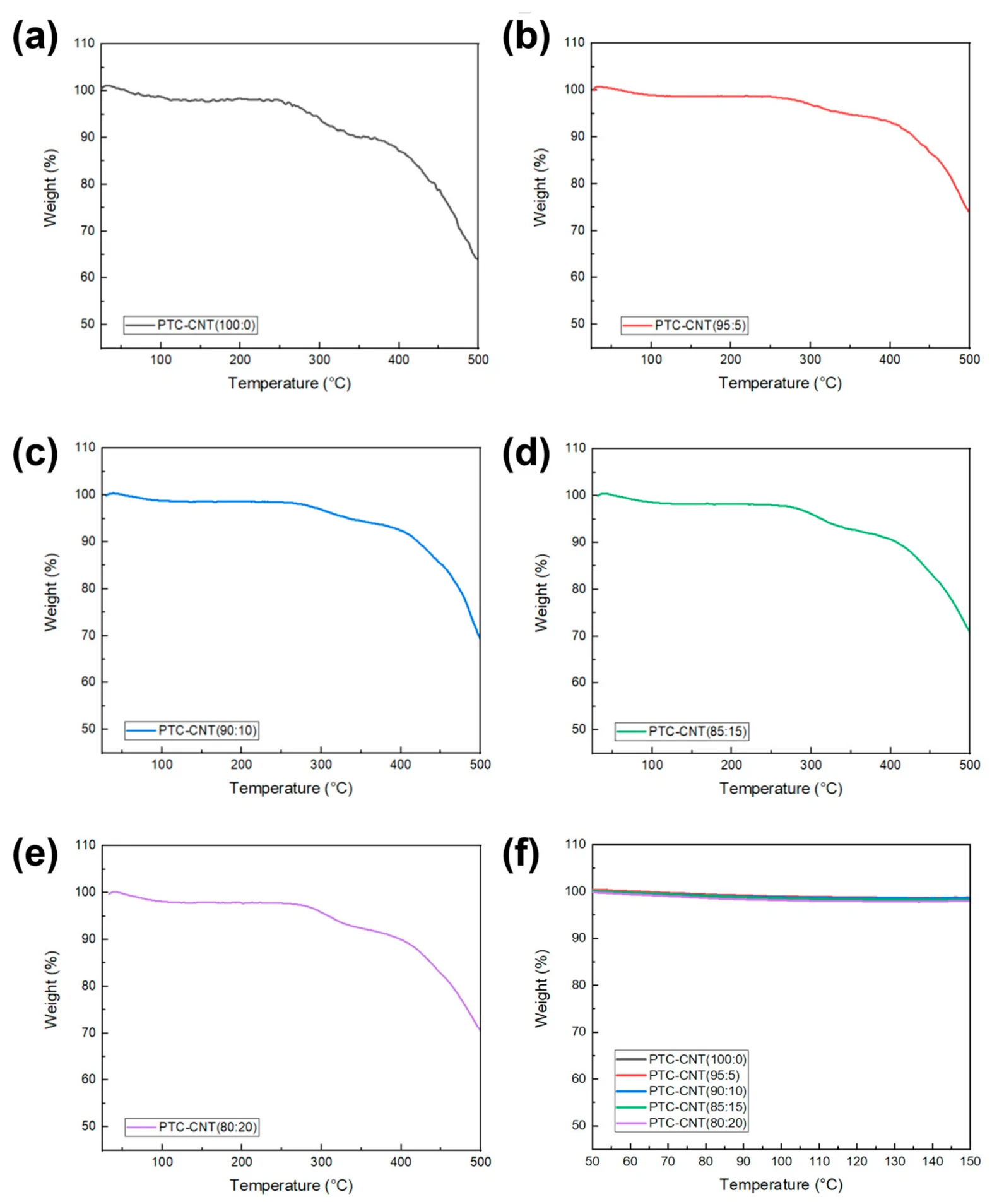
TGA curves of PTC-CNT-coated m-aramid textiles with different PTC:CNT ink ratios: (**a**) 100:0; (**b**) 95:5; (**c**) 90:10; (**d**) 85:15; (**e**) 80:20; and (**f**) enlarged comparison of the low-temperature region up to 150 °C for all compositions.

**Figure 8:**
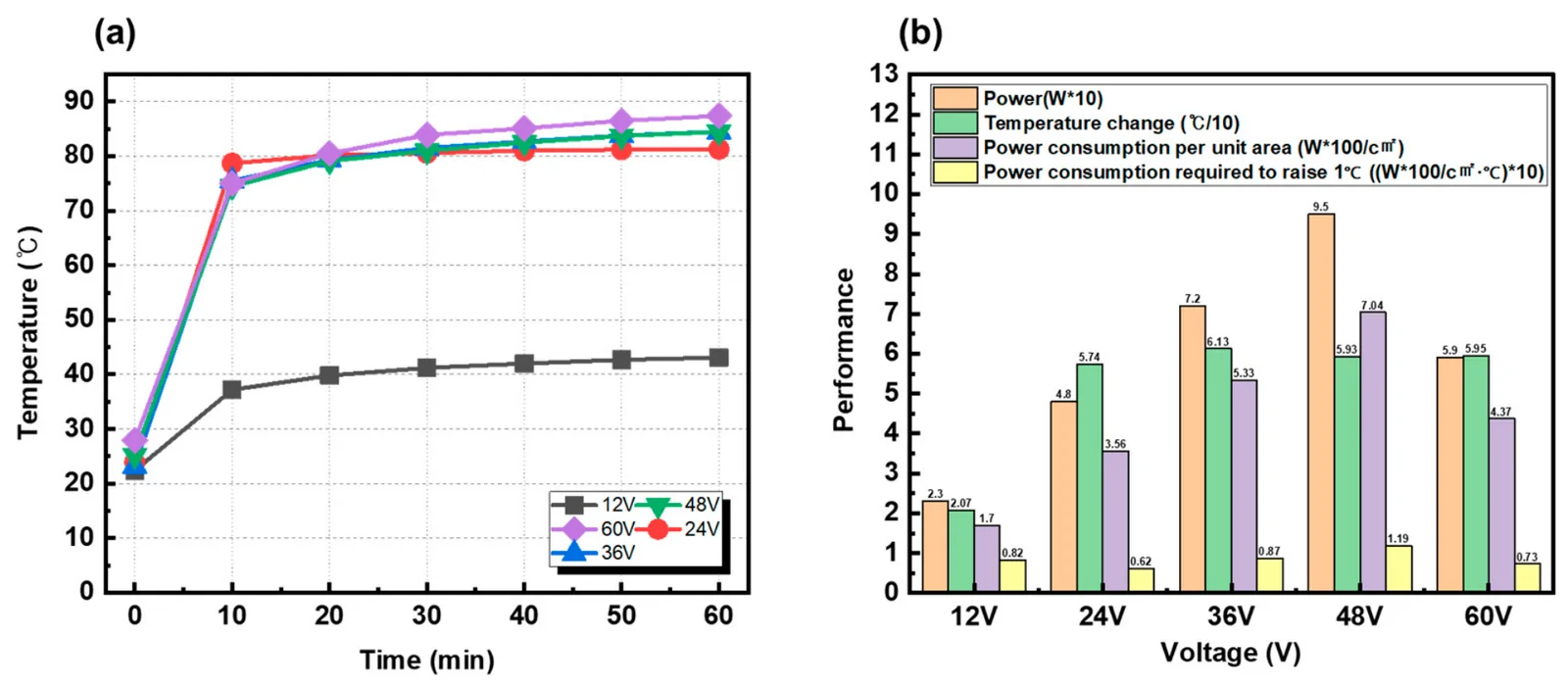
(**a**) Time–temperature curves of pure PTC105 ink under different applied voltages. (**b**) Corresponding power consumption, average TCR, power density, and energy consumption per degree of temperature rise.

**Figure 9:**
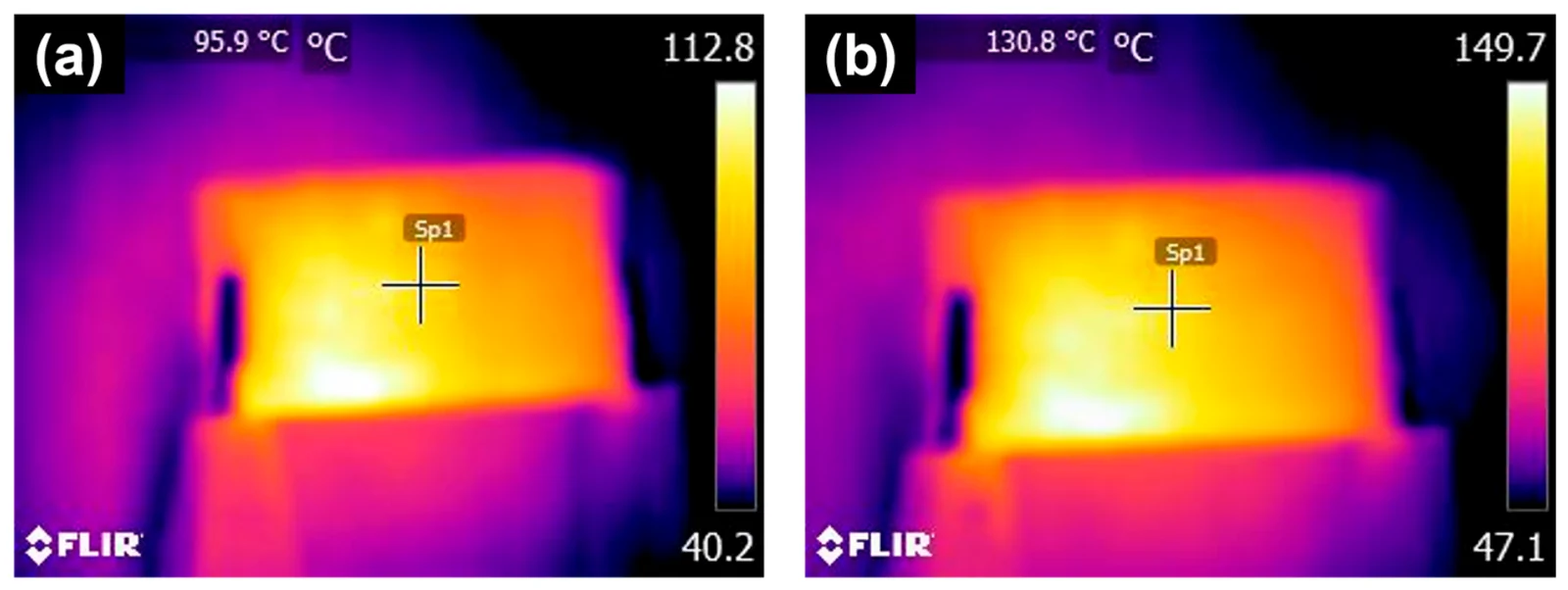
Infrared thermographic images acquired using an FLIR thermal camera for the optimized 90:10 PTC-CNT–SHT heater under a constant applied voltage of 24 V after (**a**) 1 min and (**b**) 1 h of operation. The corresponding spot temperatures were approximately 96 and 130 °C, respectively.

**Figure 10:**
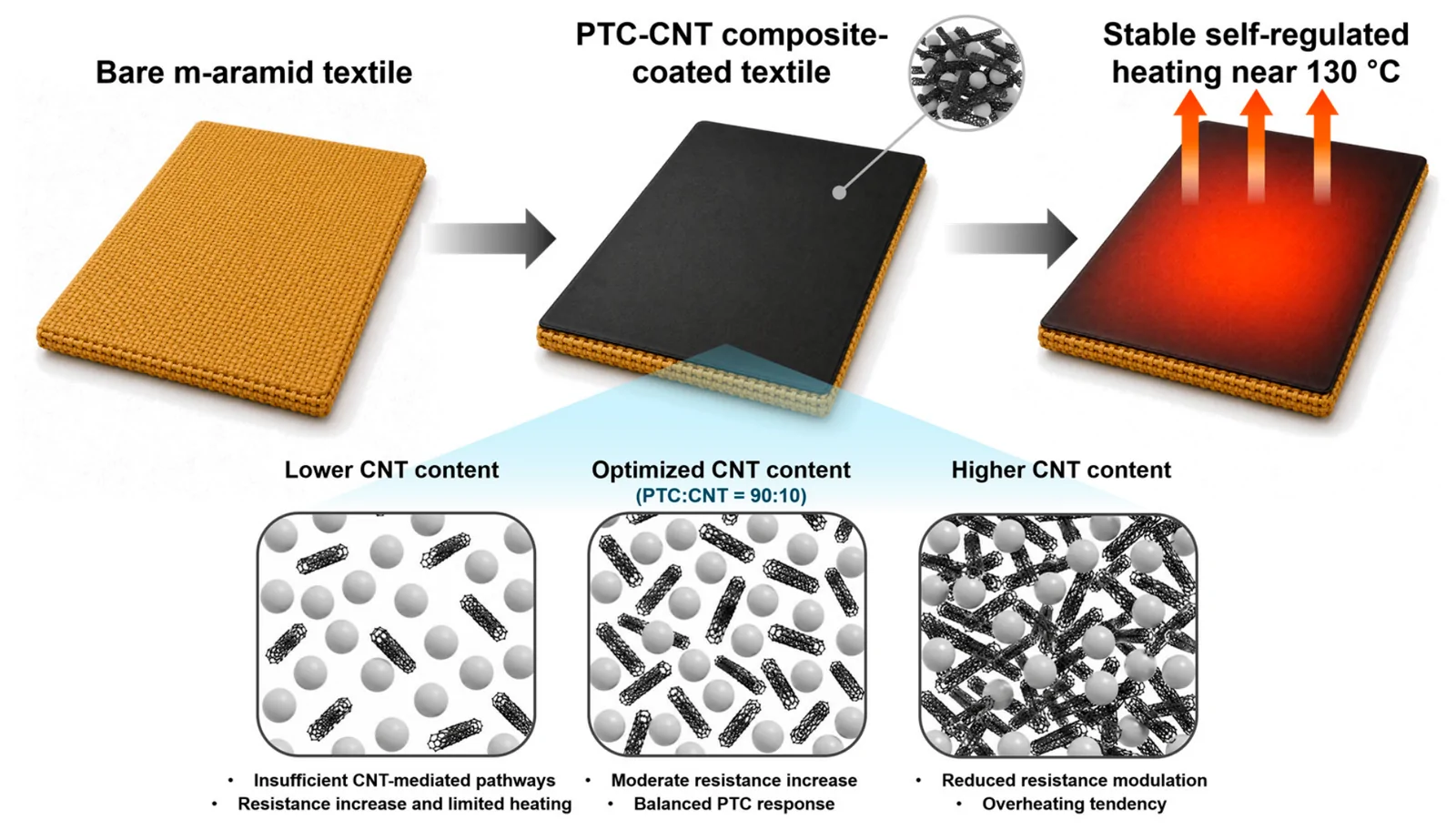
CNT-loading-dependent electrothermal behavior of the PTC-CNT textile heater.

**Figure 11:**
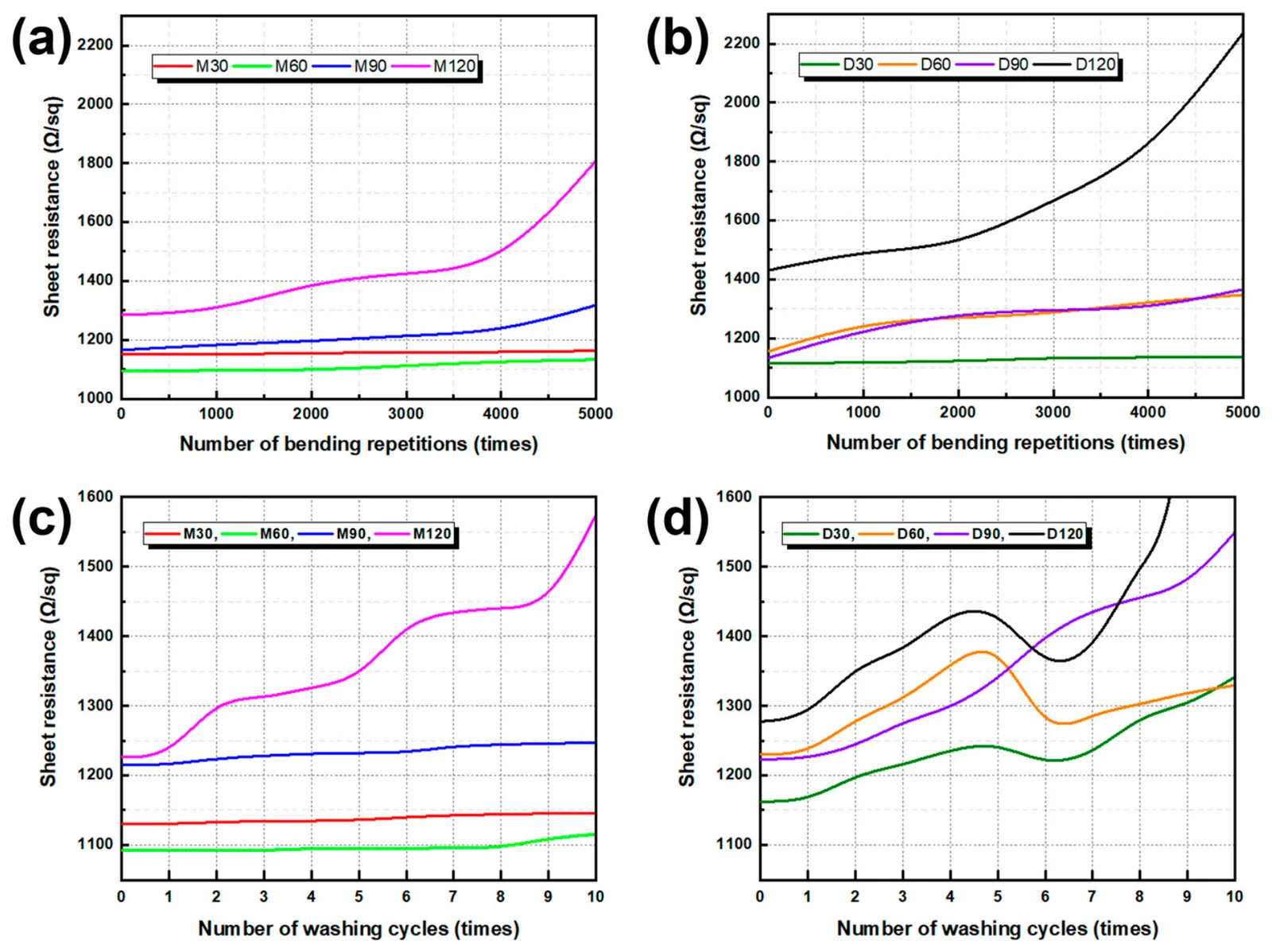
Electrical durability of PTC-CNT-coated textile heaters with different coating thicknesses. (**a**) Bending durability of m-aramid-based samples, (**b**) bending durability of Dewspo-based samples, (**c**) washing durability of m-aramid-based samples, and (**d**) washing durability of Dewspo-based samples.

**Figure 12:**
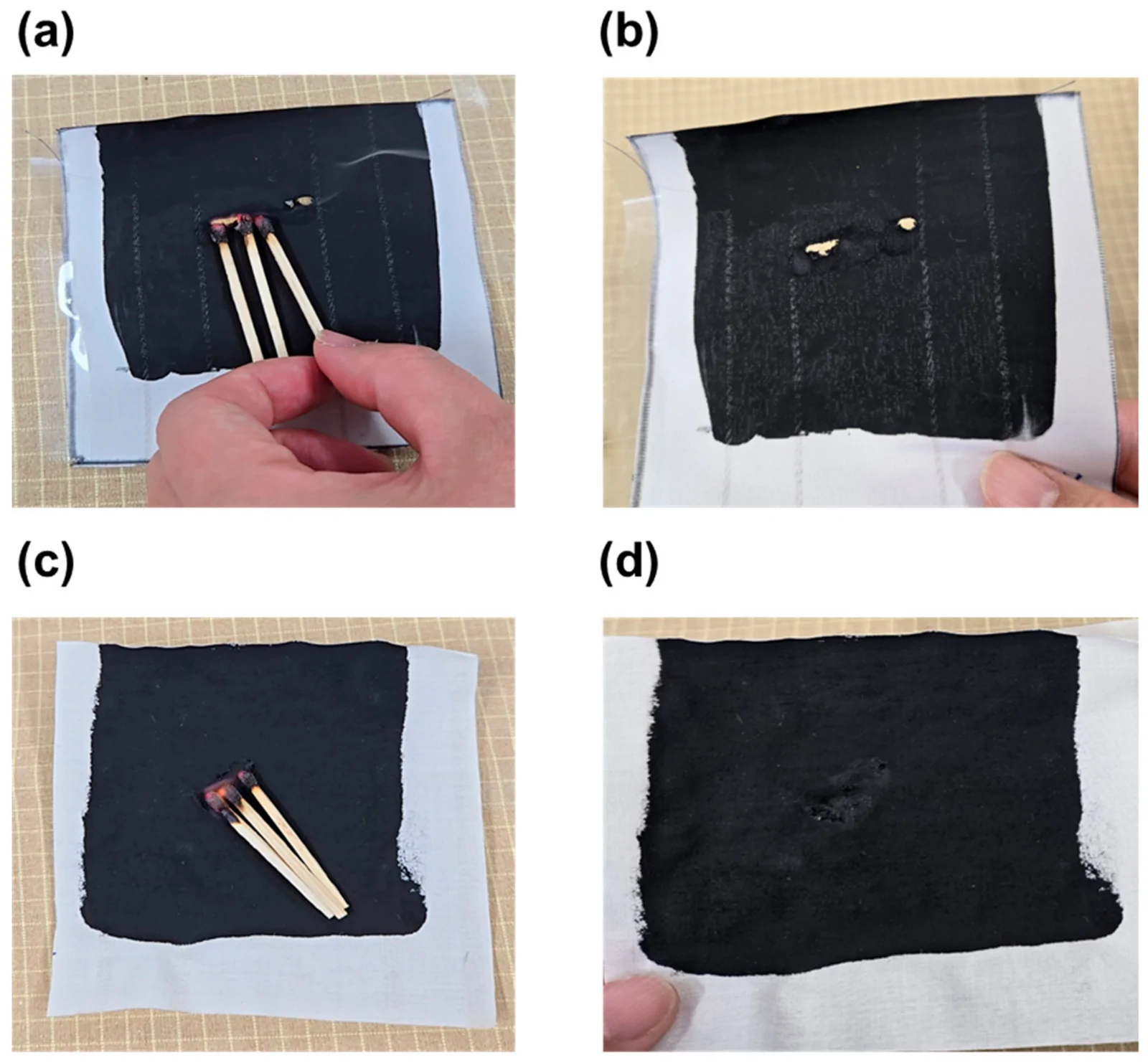
Flame-contact test for flame retardancy: (**a**,**b**) Dewspo fabric coated with PTC-CNT composite ink under and after direct flame contact. (**c**,**d**) m-aramid fabric coated with PTC-CNT composite ink under and after direct flame contact.

**Figure 13:**
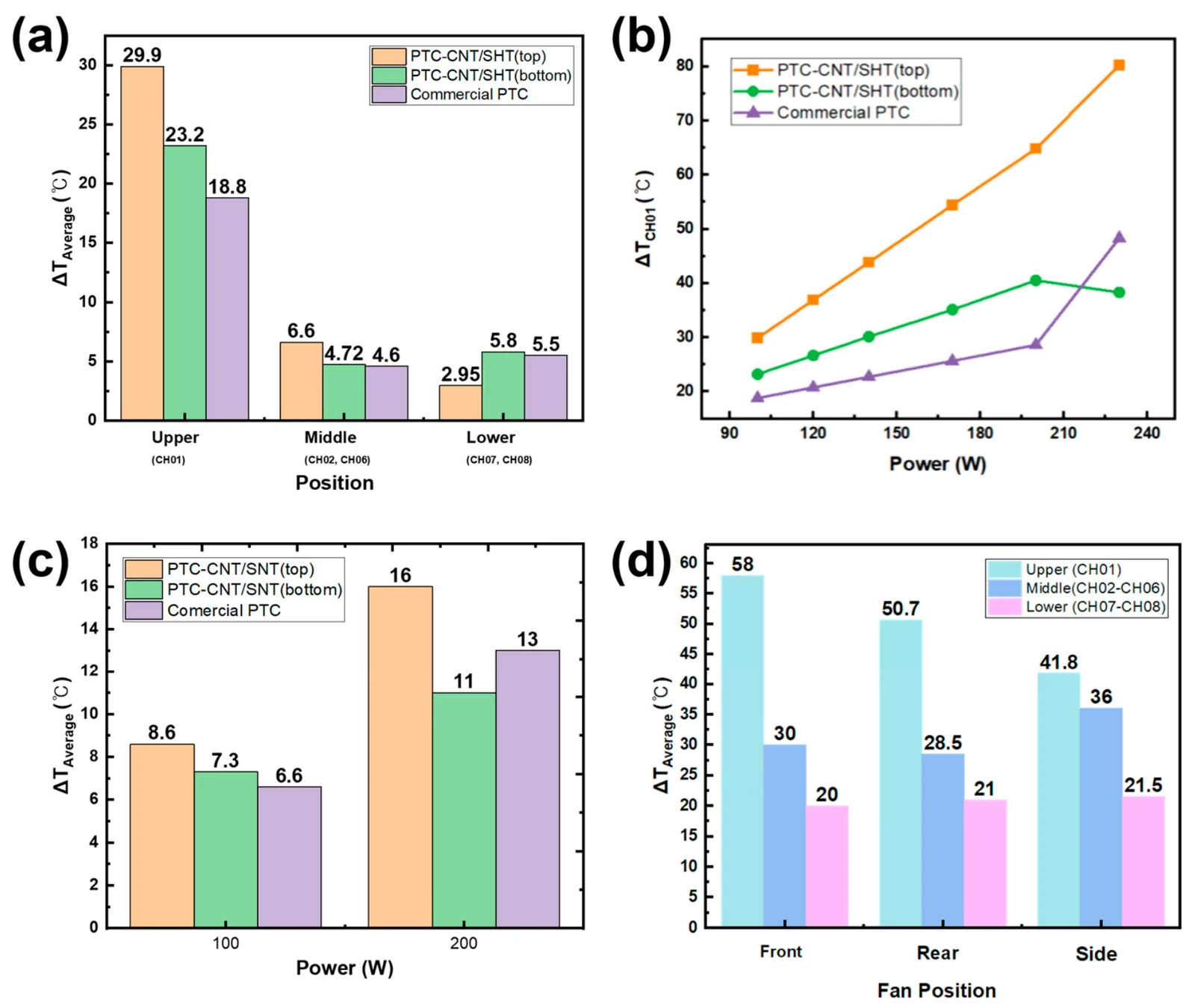
Heating performance of PTC-CNT–SHT compared with a commercial PTC heater. (**a**) Average temperature rise under 100 and 200 W conditions. (**b**) Upper-center air-temperature rise at CH01 as a function of heating power. (**c**) Vertical temperature distribution at 100 W. (**d**) Effect of fan position on temperature distribution at 240 W.

**Table 1:** Voltage-dependent heating characteristics of pure PTC105 ink (heating area 13.5 cm^2^, 1 h test).

Voltage (V)	Initial Temperature (°C)	Temperature After 1 min (°C)	Temperature After 1 h (°C)	Initial Resistance, R_1_ (Ω)	ΔR (Ω)	Power (W)	Power Density (W/m^2^)	α_avg_ (K^−1^)
12	22.4	28.5	43.1	1500	297	0.23	170	0.0096
24	23.9	62.3	81.3	1500	404	0.47	350	0.0047
36	23.2	61.9	84.5	1500	448	0.72	533	0.0049
48	25.2	54.5	85.6	1500	1069	0.95	704	0.0118
60	27.9	59.5	87.4	1500	2011	0.59	437	0.0225

**Table 2:** Electrothermal characteristics of PTC-CNT–SHTs with different PTC ratios under a 24 V input for 1 h (active heating area: 18 cm^2^).

PTC:CNT Ratio	Initial Temperature (°C)	Temperature After 1 min (°C)	Temperature After 1 h (°C)	Initial Resistance, R_1_ (Ω)	ΔR (Ω)	Power at 1 h (W)	Power Density at 1 h (W m^−2^)	α_avg_ (K^−1^)
95:5	23.4	95.6	117.4	200	601	0.72	400	0.0320
90:10	23.2	96.3	130.2	126	174	1.92	1067	0.0129
85:15	23.5	110.0	148.1	107	93	2.88	1600	0.0070
80:20	23.7	121.7	250.0	N/A	N/A	N/A	N/A	N/A

N/A: Not available owing to excessive temperature rise during the test.

**Table 3:** Comparative chamber-heating performance of PTC-CNT–SHT and commercial PTC heater under the nominal 200 W condition for 300 min.

Heater Configuration	Total Energy Consumption (Wh)	Average Power Input (W)	ΔT_avg_ (°C)	Upper-Center Air-Temperature Rise, ΔTCH01 (°C)
PTC-CNT–SHT top	994.0	198.8	16.0	64.8
PTC-CNT–SHT bottom	1001.0	200.2	11.0	40.5
Commercial PTC	1006.2	201.2	13.0	28.6

## Data Availability

The original contributions presented in this study are included in the article/Supplementary Materials. Further inquiries can be directed to the corresponding authors.

## Supplementary Materials

The following supporting information can be downloaded at https://www.mdpi.com/article/10.3390/polym18192315/s1. Figure S1. (a) Photograph of the contraction–relaxation tester with descriptions of each setting. (b) Fabric bending condition. (c) Photograph showing the actual motion of the fabric during stretching. Figure S2. (a) Fabricated samples for flame retardancy evaluation. (b) Experimental setup for voltage application. (c) Photograph of the flame retardancy test process. Figure S3. Flame-retardancy test under applied voltage: (a–c) Dewspo fabric coated with PTC-CNT–SHT, (d–f) m-aramid fabric coated with PTC-CNT–SHT. Table S1. Apparent viscosity of PTC-CNT pastes with different PTC:CNT ink ratios measured at room temperature and 60 °C at a fixed shear rate of 10 s^−1^. Data are presented as means ± standard deviation from three independent measurements (*n* = 3). Table S2. Statistical summary of the sheet resistance of PTC-CNT-coated textile heaters after cyclic bending and washing durability tests. Values are presented as means ± standard deviation based on measurements at three different positions (*n* = 3).

## Abbreviations

The following abbreviations are used in this manuscript.

EVs	electric vehicles
PTC	positive temperature coefficient
MWCNTs	multi-walled carbon nanotubes
SHT	surface-heating textile
m-aramid	meta-aramid
TCR	temperature coefficient of resistance
HVAC	heating, ventilation, and air-conditioning
CNTs	carbon nanotubes
