# Supplementary material for: Self-Regulating Polymer–Carbon Nanotube Composite Heater on Meta-Aramid Textile
Source: Polymers (Basel). 2026 Sep 22;18(19):2315. doi: 10.3390/polym18192315 (PMC13667268; doi:10.3390/polym18192315)
Supplement: Supplementary file 1 [file polymers-18-02315-s001.zip › polymers-4428450-supplementary.pdf]

Supplementary Material

Self-Regulating Polymer/CNT Composite Heater Coated on Meta-Aramid Textile with Composition-Dependent Electro-thermal Behavior

Ha Eun Kang[1,†], Ji-Hyeok Choi[1,†] , Seung Hyun Jee[2], Dong-Joo Kim[3,*], Sun Hee Kim[4,*]

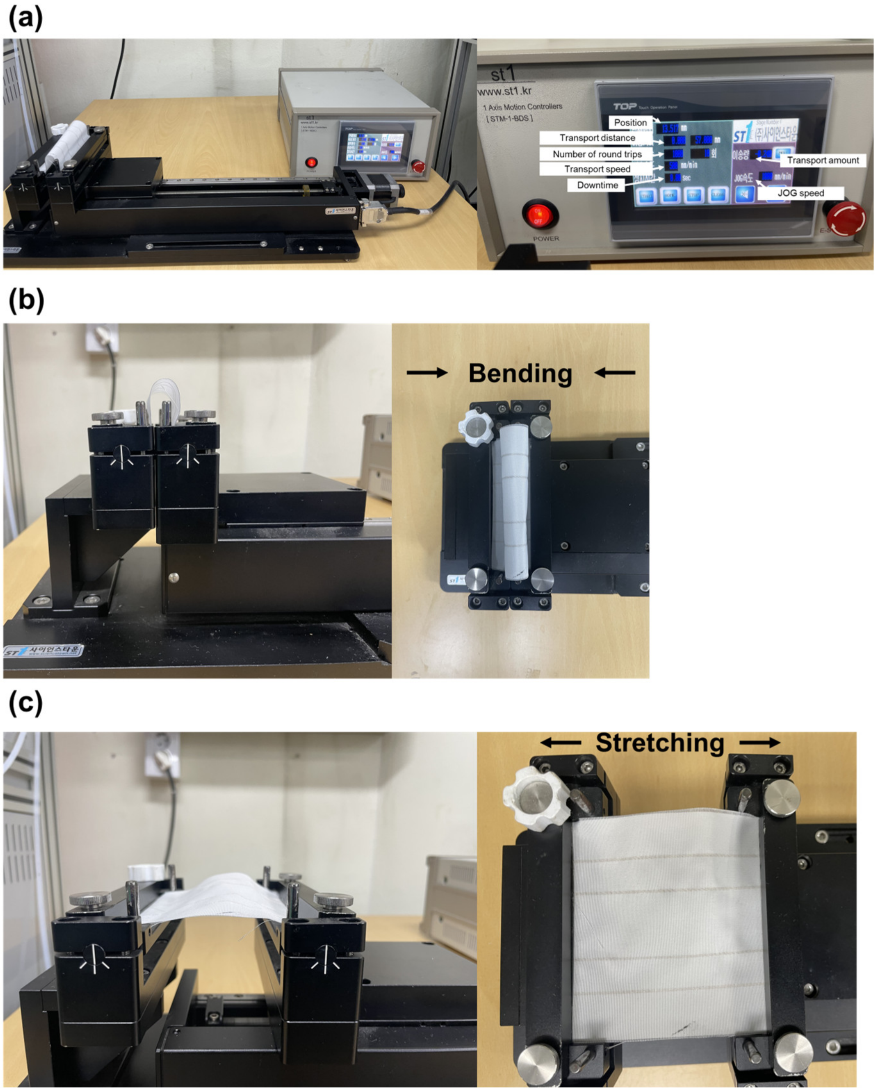

Figure S1. (a) Photograph of the contraction–relaxation tester with descriptions of each setting, (b) Fabric bending condition, (c) Photograph showing the actual motion of the fabric during stretching.

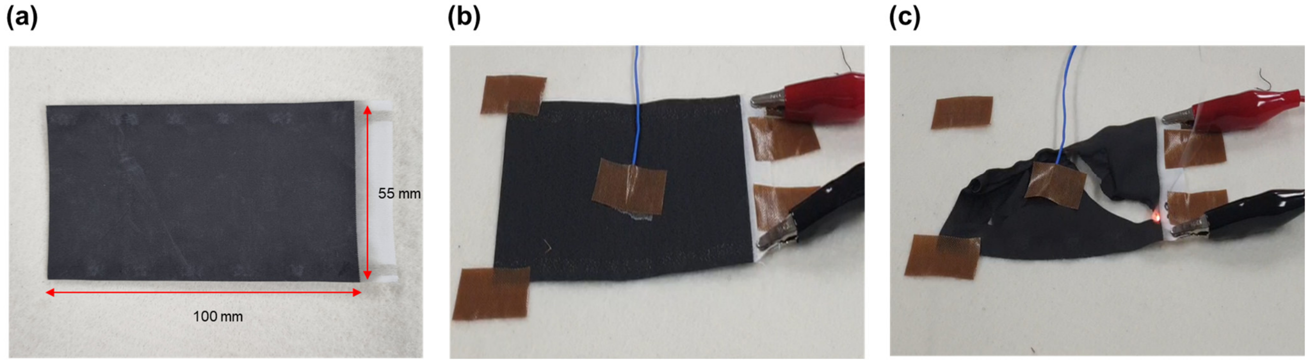

Figure S2. (a) Fabricated samples for flame retardancy evaluation, (b) Experimental setup for voltage application, (c) Photograph of the flame retardancy test process.

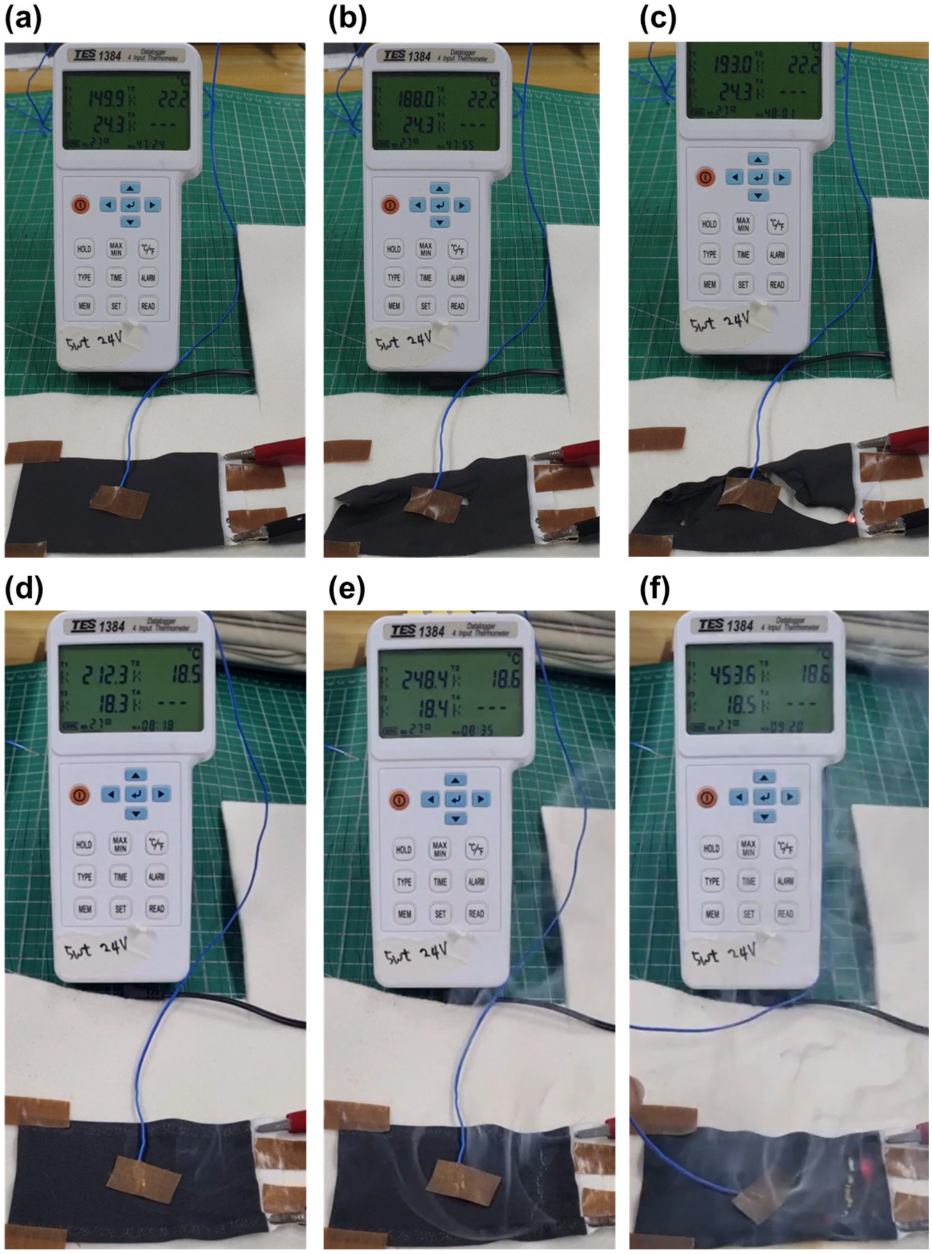

Figure S3. Flame-retardancy test under applied voltage: (a)-(c) Dewspo fabric coated with PTC-CNT SHT, (d-f) m-aramid fabric coated with PTC-CNT/SHT.

Table S1. Apparent viscosity of PTC-CNT pastes with different PTC:CNT ink ratios measured at room temperature and 60 °C at a fixed shear rate of 10 s^{-1}. Data are presented as mean ± standard deviation from three independent measurements (n = 3).

PTC:CNT ink ratio	Viscosity at RT (cP)	Viscosity at 60 °C (cP)
100:00:00	21,950 ± 238	8,781 ± 77
95:05:00	15,190 ± 157	5,099 ± 56
90:10:00	13,790 ± 116	6,147 ± 70
85:15:00	12,760 ± 120	4,274 ± 49
80:20:00	11,520 ± 89	3,687 ± 34

Table S2. Statistical summary of the sheet resistance of PTC-CNT-coated textile heaters after cyclic bending and washing durability tests. Values are presented as mean ± standard deviation based on measurements at three different positions (n = 3).

Sample	Sheet resistance after 5000 bending cycles (Ω/sq)	Sheet resistance after 10 washing cycles (Ω/sq)
M30	1163±32.5	1146±34.4
M60	1132±33.5	1116±34.0
M90	1317±34.9	1245±39.5
M120	1808±39.2	1574±46.2
D30	1136±31.2	1341.4±39.7
D60	1347±41.9	1329.5±40.0
D90	1366±44.7	1549.9±45.5
D120	2237±45.9	2870.5±48.1
